# The Up-Regulation of Oxidative Stress as a Potential Mechanism of Novel MAO-B Inhibitors for Glioblastoma Treatment

**DOI:** 10.3390/molecules24102005

**Published:** 2019-05-25

**Authors:** Guya Diletta Marconi, Marialucia Gallorini, Simone Carradori, Paolo Guglielmi, Amelia Cataldi, Susi Zara

**Affiliations:** 1 Department of Pharmacy, University “G. d’Annunzio” of Chieti-Pescara, Via dei Vestini 31, 66100 Chieti, Italy; guya.marconi@virgilio.it (G.D.M.); marialucia.gallorini@unich.it (M.G.); amelia.cataldi@unich.it (A.C.); susi.zara@unich.it (S.Z.); 2 Department of Drug Chemistry and Technologies, Sapienza University of Rome, P.le A. Moro 5, 00185 Rome, Italy; paolo.guglielmi@uniroma1.it

**Keywords:** Glioblastoma, MAO-B inhibitors, oxidative stress, migration

## Abstract

Gliomas are malignant brain tumors characterized by rapid spread and growth into neighboring tissues and graded I–IV by the World Health Organization. Glioblastoma is the fastest growing and most devastating IV glioma. The aim of this paper is to evaluate the biological effects of two potent and selective Monoamine Oxidase B (MAO-B) inhibitors, Cmp**3** and Cmp**5**, in C6 glioma cells and in CTX/TNA2 astrocytes in terms of cell proliferation, apoptosis occurrence, inflammatory events and cell migration. These compounds decrease C6 glioma cells viability sparing normal astrocytes. Cell cycle analysis, the Mitochondrial Membrane Potential (MMP) and Reactive Oxygen Species (ROS) production were detected, revealing that Cmp**3** and Cmp**5** induce a G1 or G2/M cell cycle arrest, as well as a MMP depolarization and an overproduction of ROS; moreover, they inhibit the expression level of inducible nitric oxide synthase 2, thus contributing to fatal drug-induced oxidative stress. Cmp**5** notably reduces glioma cell migration via down-regulating Matrix Metalloproteinases 2 and 9. This study demonstrated that our novel MAO-B inhibitors increase the oxidative stress level resulting in a cell cycle arrest and markedly reduces glioma cells migration thus reinforcing the hypothesis of a critical role-played by MAO-B in mediating oncogenesis in high-grade gliomas.

## 1. Introduction

Gliomas are the first brain tumors assumed to originate from the neuroglial stem or from progenitor cells, characterized by inappropriate proliferation, infiltration into neighboring normal brain tissue and the disruption of the normal functioning of the brain. Based on their histological appearance, gliomas are conventionally classified as astrocytic, oligodendroglial or ependymal tumors and attributed by the World Health Organization grades I–IV, which denote disparate levels of malignancy [1]. Glioblastoma (GBM), a grade IV astrocytoma notoriously treatment–resistant brain cancer, is the greatest destructive brain tumor, largely due to the limited effects of conventional post-surgical chemotherapeutic agents and to irradiation [2].

A better understanding of the mechanism underlying the destructive nature of GBM could improve radio-, chemo- and gene therapy. More effective GBM therapeutic strategies are urgently needed. Unfortunately, the present therapies for high-grade gliomas are not an effective approach; after preliminary diagnosis, patients receive a maximal surgical resection, concomitant radiotherapy and chemotherapy with Temozolomide (TMZ), the currently used chemoterapeutic agent for GBM treatment. The TMZ raised the prognosis of GBM patients because it can cross the blood-brain barrier and promote glioma cell death by alkylating DNA, even if TMZ resistance could be one of the major reasons why treatments are not effective [3].

Monoamine Oxidase (MAO) catalyzes the oxidative deamination of a range of monoamines, including 5-hydroxytryptamine (5-HT, serotonin), histamine and the catecholamines dopamine, noradrenaline and adrenaline. The two isoenzymes of MAO (MAO-A and MAO-B) are present in most mammalian tissues. They are not uniformly distributed in the human brain, and the principal form in the basal ganglia is MAO-B. Over the past several decades, MAO-B inhibitors showed to have potential use in the treatment of several neurodegenerative disorders, including Parkinson’s disease and Alzheimer’s disease [4].

**Figure 1:**
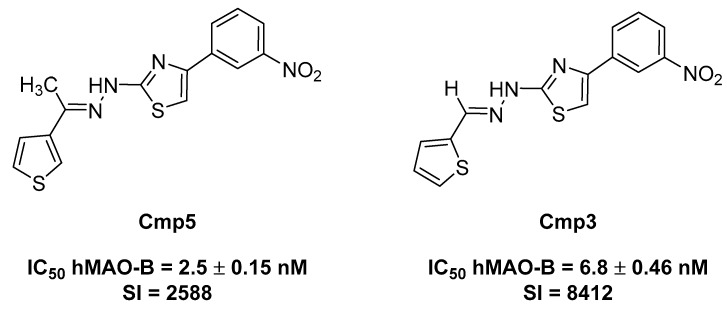
Structures and monoamine oxidase (MAO) inhibitory activity of the tested compounds.

However, in the paper of Gabilondo et al. [5], it was reported that a remarkable and selective increase in MAO-B activity is also detected in human gliomas. This could be explained by an augmented number of astrocytes as well as a higher content of MAO-B in tumoral astrocytes with respect to normal ones [5], highlighting that the overexpression of MAO-B could be related with high-grade glioma, and thus defining MAO-B as a promising target for the development of novel therapies for cancer. To better understand the role played by MAO-B inhibitors in the treatment of glioma, the aim is to evaluate two newly synthesized compounds, namely Cmp**3** and Cmp**5** (Figure 1) [6], along with two approved selective MAO-B inhibitors (deprenyl and safinamide). These compounds were the best-in-class derivatives, within the hydrazothiazole scaffold, endowed with a potent and selective hMAO-B inhibition in the low nanomolar range comparable to the clinically used drugs. The Selectivity Index (SI) was expressed as IC_50_ hMAO-A/IC_50_ hMAO-B. They were shown to act as reversible and competitive inhibitors. We also assessed the in vitro stability of pH and temperature in forced conditions [7].

The aim of this work is to investigate the biological response of rat C6 glioma cell line and CTX TNA2 astrocytes after treatment with these two novel MAO-B inhibitors in terms of cell proliferation, apoptosis occurrence, inflammatory events and cell migration.

## 2. Results

### 2.1. Effects of MAO-B Inhibitors on Rat C6 Glioma and on CTX TNA2 Astrocytes Cell Viability

Two novel, selective and potent MAO-B inhibitors, Compound **3** (Cmp**3**) and Compound **5** (Cmp**5**), were selected and kept for further investigations (Figure 1). The effects of Cmp**3** and Cmp**5** were evaluated by means of MTT assay on rat C6 glioma cell line compared with non-tumoral CTX TNA2 astrocytes. Cmp**3** and Cmp**5** were tested individually and compared to TMZ, the widely used drug in GBM treatment, to evaluate the effect on cell viability.

**Figure 2:**
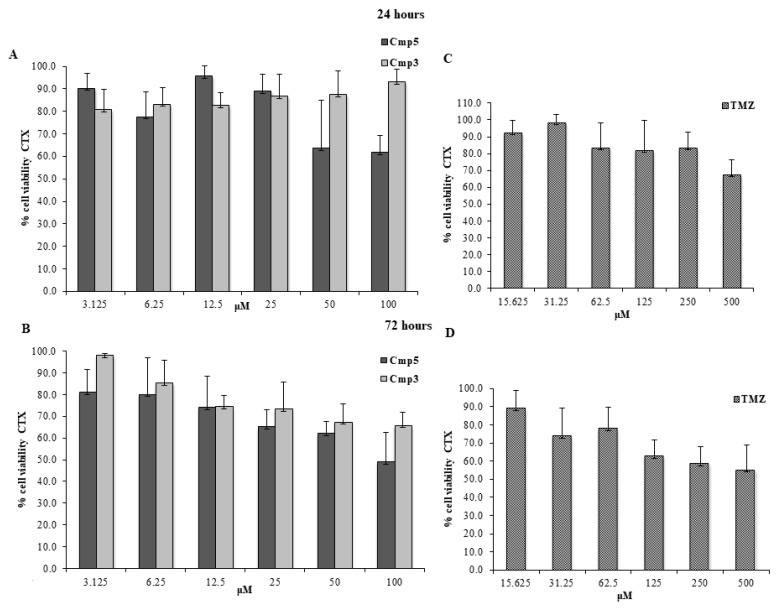
MTT cell viability assay on CTX. (**A,B**) Histograms represent the viability dose-response of CTX cells exposed to different concentrations of Cmp**3** and Cmp**5** (from 3.125 to 100 μM) for 24 h and 72 h, respectively. (**C,D**) Histograms represent the viability dose-response of CTX cells exposed to different concentrations of TMZ (from 15.636 to 500 μM) for 24 h and 72 h. Proliferation was assessed using MTT assay and normalized to control cells treated with DMSO (0.2% as final concentration).

**Figure 3:**
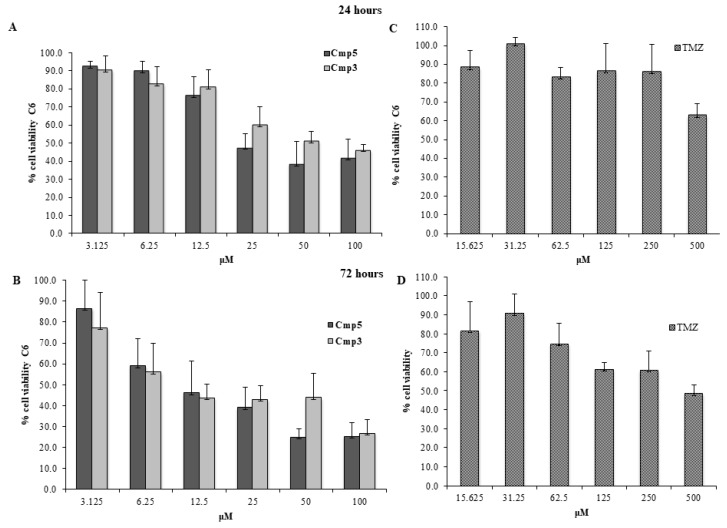
MTT cell viability assay on C6. (**A,B**) Histograms represent the viability dose-response of C6 cells exposed to different concentrations of Cmp**3** and Cmp**5** (from 3.125 to 100 μM) for 24 h and 72 h. (**C,D**) Histograms represent the viability dose-response of C6 cells exposed to different concentrations of TMZ (from 15.636 to 500 μM) for 24 h and 72 h. Proliferation was assessed using MTT assay and normalized to control cells treated with DMSO (0.2% as final concentration).

Cmp**3** and Cmp**5** were administered at doses ranging from 3.125 up to 100 μM, whereas TMZ was administered at doses ranging from 15.625 up to 500 μM, based on the results already reported in literature [8,9]. In CTX TNA2 astrocytes, Cmp**5** at 100 μM evidences cell viability percentages of ~60% and ~50% after 24 h (Figure 2A) and 72 h of treatment (Figure 2B), respectively, whereas Cmp**3** at 100 μM does not affect the cell viability after 24 h (Figure 2A) and displays a cell viability percentage of ~60% after 72 h (Figure 2B) of treatment. Conversely, TMZ at 500 μM is shown to present cell viability percentages of ~70% and ~60% after 24 h (Figure 2C) and 72 h of treatment (Figure 2D), respectively. Cmp**3** and Cmp**5** drastically reduce the viable C6 cells at doses between 50 and 100 μM after 24 h and 72 h of treatment (Figure 3A,B, respectively), while TMZ at 500 μM shows similar C6 cell viability percentages reported for CTX TNA2 at the same time points. (Figure 3C,D, respectively). These results lead us to keep 50 μM for the Cmp**5** and 100 μM for the Cmp**3** as suitable doses for further investigations.

For the sake of comparison, we also tested for the first time two well-recognized, potent and selective hMAO-B inhibitors (deprenyl and safinamide) in order to better corroborate our proof-of-concept. Our newly synthesized compounds (Cmp**3** and Cmp**5**) were nanomolar inhibitors of hMAO-B working in the same range of these two approved drugs. Preliminary MTT experiments regarding these two approved hMAO-B inhibitors against C6 and CTX TNA2 cells at 24 h and 72 h (Figure S1) assessed that, at lower concentrations (0.625–10 µM), safinamide and deprenyl display no statistically significant differences between tumoral and non-tumoral cell lines (a biological effect which can be further stressed at higher concentrations), differently from what is shown by our novel compounds (Cmp**3** and Cmp**5**).

### 2.2. Influence of Cmp**3** and Cmp**5** on Programmed Cell Death and Necrosis

**Figure 4:**
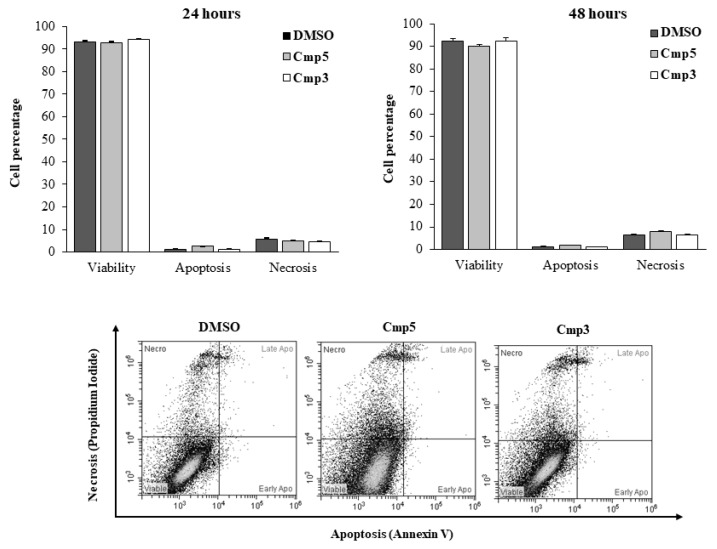
Induction of apoptosis and necrosis in C6 cells in the presence of Cmp**5** and Cmp**3**. Percentages of cells found in the various phases of cell death, analyzed by flow cytometry after staining with Annexin V-FITC and propidium iodide (PI), are shown in the upper panel. Bars represent the percentage of viable cells (unstained), cells in apoptosis (Annexin V-positive), cells in late apoptosis (Annexin V+PI), and cells in necrosis (PI) as medians ± SD calculated from three independent experiments. Representative dual-parameter fluorescence density dot plots at 48 h are displayed in the lower panel.

The influence of MAO-B inhibitors on the induction of apoptosis in the C6 rat glioma cell cultures after 24 h and 48 h of treatment is shown in Figure 4. DMSO-treated cells remain viable over the time of the culture, being the percentage of cells stained negative for both Annexin-V and PI assessed at 92.94% and 92.28%, after 24 h and 48 h, respectively. As for cells exposed to Cmp**3**, the percentage of viable cells is comparable to that detected for DMSO both after 24 h (94.30%) and 48 h (92.47%). Apoptosis and/or necrosis are not observed. A slight, but not significant decrease in the viability of cells exposed to the Cmp**5** can be established after 48 h of treatment (90.07%) as well as a rise in the percentage of the PI-stained necrotic population (8.05%) compared to that of DMSO-treated cells (6.47%).

### 2.3. Regulation of the Cell Cycle in the C6 Cells Exposed to Cmp**3** and Cmp**5**

**Figure 5:**
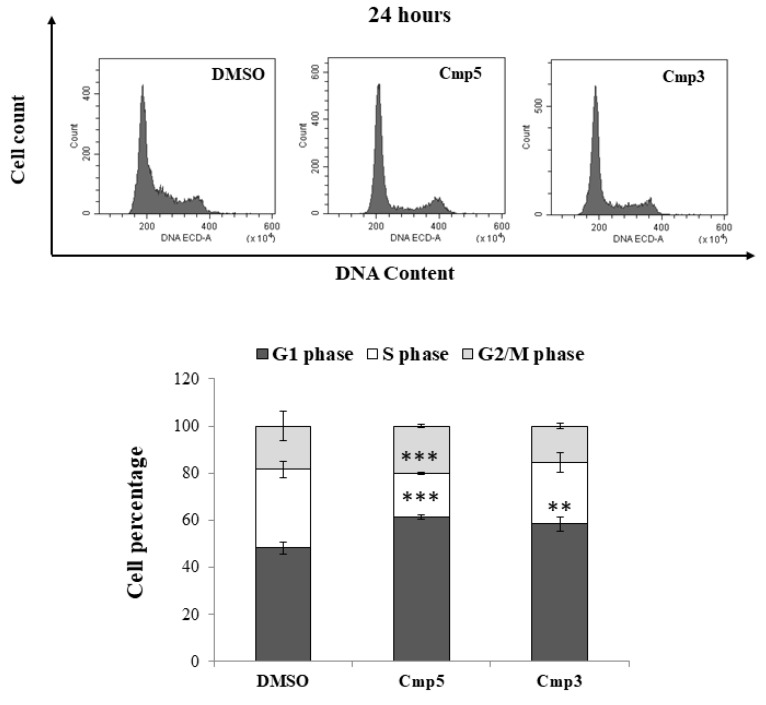
Cell cycle analysis in C6 rat glioma cells in the presence of Cmp**5** and Cmp**3**. The upper panel displays the different DNA profile of cells after a 24 h treatment. Peaks are generated by the emission of PI in the FL3 fluorescence channel. The percentage of cells in the G1, S and G2 phases are shown in the bar graph in the lower panel. Bars represent medians ± SD from three independent experiments. *** *p* < 0.002 between G1 phase of DMSO and Cmp**5**; *** *p* < 0.002 between S phase of DMSO and Cmp**5**; ** *p* < 0.02 between G1 phase of DMSO and Cmp**3**.

In order to establish whether the two MAO-B inhibitors could induce cell cycle arrest checkpoints and decrease the C6 cells proliferation, the DNA content profiles of cultures exposed to the Cmp**3** and Cmp**5** after 24 h (Figure 5, upper panel) obtaining percentages of cells found in G1, S and G2 phase (Figure 5, lower panel) were analyzed. The DNA content fluorescence emission peaks and bars related to the DMSO sample display a typical cell cycle profile, with proliferative and active cells (G1 phase = 48.18%; S phase = 33.46%; G2/M phase = 18.37%). The Cmp**3** causes a delay of the cell cycle in the G1 phase, being the percentage of cells increased in respect to DMSO-exposed cells (58.46%). Likewise, the percentage of cells found in the G1 phase after the Cmp**5** administration is even higher, being assessed at 61.51%. In parallel, a consistent and remarkable change in the percentage of cells found in the S phase is registered in the presence of the Cmp**3** (26.27%) and is further enhanced with the Cmp**5** (18.34%).

### 2.4. Generation of Reactive Oxygen Species (ROS) and Depolarization of the Mitochondrial Membrane Potential (MMP) in Cells Exposed to Cmp**3** and Cmp**5**

**Figure 6:**
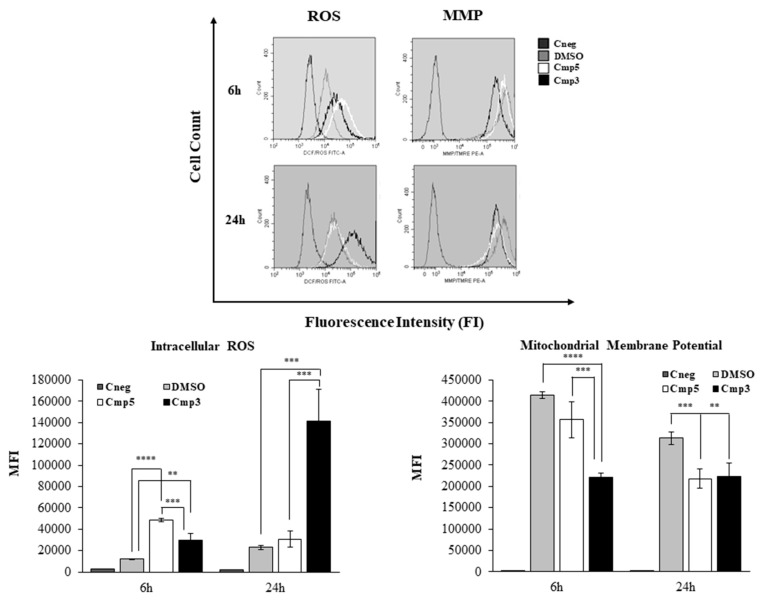
Generation of intracellular reactive oxygen species (ROS) and mitochondrial membrane potential (MMP) modulation in C6 cells in the presence of Cmp**5** and Cmp**3**. Bars in the lower panel represent median values ± SD of the mean fluorescence intensity (MFI) generated by the oxidation of CM-H2DCF-DA (generation of intracellular ROS) and by the emission of TMRE (MMP) measured by flow cytometry in cells exposed to MAO-B inhibitors. Representative fluorescence emission peaks are shown in the upper panel and are provided to display the shift in the fluorescence emissions in the FL1 (FITC) and FL2 (PE) channels. **** *p* < 0.0005, *** *p* < 0.005, ** *p* < 0.02.

Oxidative stress, as detected by the oxidation of CM-H2DCF-DA, significantly increases when the C6 cells are exposed to Cmp**3** and Cmp**5** after 6 h (Figure 6). Both Cmp**3** and Cmp**5** generate ROS, registering a 2.4- (Cmp**3**) and a 4-fold (Cmp**5**) increase in the DCF fluorescence intensity compared to DMSO-treated culture. After a 24 h exposure, the Cmp**3** dramatically rises the ROS production, with a 6.2-fold increase in respect to cells exposed to DMSO while the DCF levels related to Cmp**5**-exposed culture are comparable with the one exposed to DMSO. According to the induction of oxidative stress, MMP is found depolarized in the presence of the two MAO inhibitors as shown in Figure 6. In more detail, after 6 h treatment Cmp**3** halves the MMP as compared to exposure to DMSO control. The depolarization of the MMP caused by the Cmp**3** exposure is remarkable as compared to MMP depolarization upon Cmp**5** treatment after the same exposure period, being the MMP level comparable to the DMSO sample.

After longer experimental times (24 h), Cmp**3** retains a consistent and significant disturbance of the MMP, in respect to the DMSO sample, being Mean Fluorescence Intensities (MFIs) assessed at 2.23 × 10^5^ (Cmp**3**) and 3.13 × 10^5^ (DMSO). In parallel, Cmp**5** considerably lowers MMP if compared to 6 h exposure, revealing values comparable with those registered for Cmp**3** (MFI of Cmp**5** = 2.18 × 10^5^) (Figure 6).

### 2.5. Nitric Oxide Synthase 1 (NOS-1), Nitric Oxide Synthase 2 (NOS-2) and Vascular Endothelial Growth Factor (VEGF) Expression in Response to MAO-B Inhibitors in Rat C6 cells

**Figure 7:**
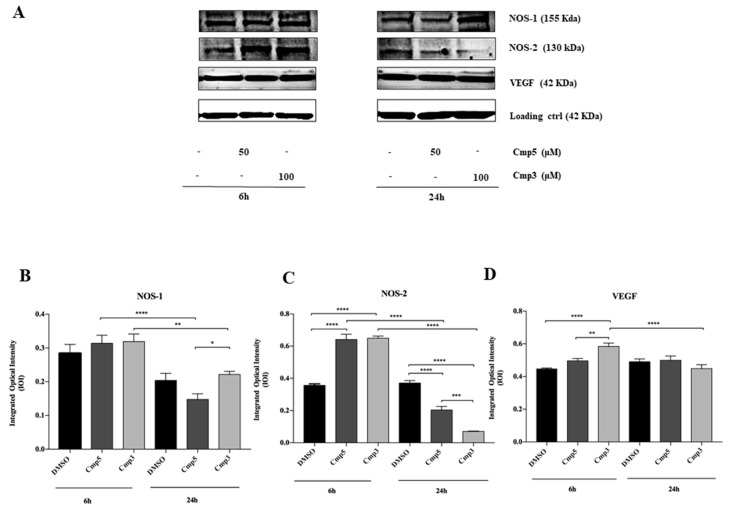
Western blotting analysis of NOS-1, NOS-2 and VEGF expression in rat C6 glioma cell lines treated with Cmp**5** and Cmp**3**. (**A**) Cells treated with DMSO (0.2%) were loaded as the negative control. Each membrane has been probed with β–actin antibody to verify loading consistency. Western blot is the most representative of three different experiments. (**B**–**D**) Histograms represent densitometric measurements of proteins bands expressed as integrated optical intensity (IOI) mean of three separate experiments. The error bars on these graphs show standard deviation (± SD). Densitometric values analysed by ANOVA (post hoc application of Tukey’s multiple comparison test) return significant differences. **** *p* < 0.0001, *** *p* < 0.0002, ** *p* < 0.0005, * *p* < 0.005.

To identify the effects of Cmp**3** at 100 μM and Cmp**5** at 50 μM on the inflammatory event induction, a Western Blot Analysis of neuronal NOS-1 and inducible NOS-2 was performed after 6 and 24 h of treatment. After 6 h of treatment, no significant difference in NOS-1 expression level is recorded in samples treated with both Cmp**3** and Cmp**5** with respect to the DMSO sample. After 24 h of treatment, the NOS-1 expression level is significantly lower in cells treated with Cmp**5** in respect to cells treated with Cmp**3**. Moreover, from 6 h to 24 h of treatment, a statistically significant decrease of the NOS-1 expression is detectable for Cmp**3** and Cmp**5** (Figure 7A,B).

After 6 h of treatment a statistically significant increase in the NOS-2 expression level is appreciable in samples treated with both Cmp**3** and Cmp**5** with respect to DMSO sample. After 24 h of treatment the NOS-2 expression is markedly reduced in cells treated with both Cmp**3** and Cmp**5** in respect to the DMSO sample. A notable decrease of the protein expression in samples, exposed to both Cmp**3** and Cmp**5**, is reported from 6 h to 24 h of exposure (Figure 7A,C).

Furthermore, a western blot of VEGF, a major contributor of tumorigenesis and angiogenesis, was performed. After 6 h, the protein is significantly higher in cells treated with Cmp**3** in respect to the DMSO sample; conversely, no significant differences are found in C6 cells treated with Cmp**5** as compared to DMSO. In addition, a significant reduction of the VEGF expression is evidenced for cells treated with Cmp**3** from 6 to 24 h (Figure 7A,D).

### 2.6. Prostaglandin E2 (PGE-2) Secretion Level in Cells Exposed to Cmp**3** and to Cmp**5**

**Figure 8:**
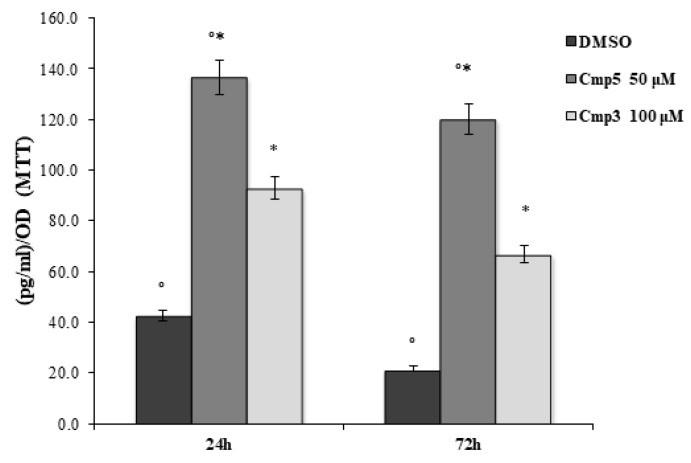
ELISA assay in C6 rat glioma cells in the presence of Cmp**3** and Cmp**5**. PGE2 secretion levels, measured after 24 and 72 h, are reported as pg/mL/OD MTT values. Data shown are the mean (± SD) of three different experiments. Cmp**5** 50 μM vs. ctrl, *Cmp**3** 100 μM vs. Cmp**5** 50 μM *p* < 0.05.

The ELISA assay for PGE-2 secretion was performed on the C6 cell line treated with Cmp**5** and Cmp**3** at 50 μM and 100 μM, respectively, for 24 and 72 h (Figure 8). As evidenced in Figure 8, both compounds notably enhanced the secretion of PGE-2 on rat C6 glioma cell lines at 24 h and 72 h compared to cells treated with DMSO. Furthermore, Cmp**5** at 50 μM is responsible for a higher PGE-2 secretion level with respect to cells treated with Cmp**3** at 100 μM after 24 and 72 h.

### 2.7. Effects of MAO-B Inhibitors on Cell Migration and Metalloproteinases Expression

**Figure 9:**
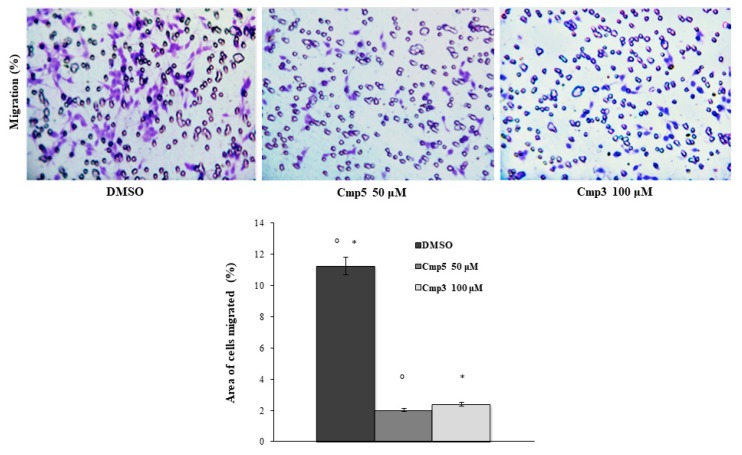
Transwell migration assay in C6 rat glioma cell line in the presence of Cmp**5** and Cmp**3**. Dose treatments are 50 and 100 μM for Cmp**5** and Cmp**3**, respectively (upper panel). Histogram represents densitometric analysis determined by quantifying thresholded area for violet color in ten fields for each of three slides per sample (lower panel). Data are presented as mean ± standard deviation. Cmp**5** 50 μM versus ctrl; *Cmp**3** 100 μM versus ctrl: *p* < 0.0001.

**Figure 10:**
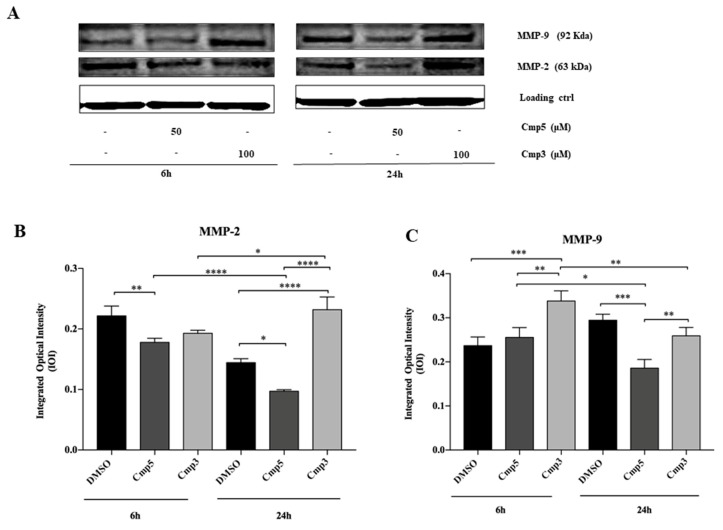
Western blotting analysis of MMP-2, MMP-9 expression in rat C6 glioma cell lines treated with Cmp**5** and Cmp**3**. (**A**) Cells treated with DMSO (0.2%) were loaded as negative control. Each membrane has been probed with β–actin antibody to verify loading consistency. Western blot is the most representative of three different experiments. (**B**,**C**) Histograms represent densitometric measurements of proteins bands expressed as integrated optical intensity (IOI) mean of three separate experiments. The error bars on these graphs show standard deviation (± SD). **** *p* < 0.0001, *** *p* < 0.0002, ** *p* < 0.0005, * *p* < 0.005.

A transwell migration assay was executed using an 8 µm pore size polycarbonate membrane in C6 cells in the absence or presence of 100 μM Cmp**3** and 50 μM Cmp**5**. Cells were exposed for 24 h to medium with or without chemoattractant (Foetal Bovine Serum) in the presence or absence of compounds where migrated cells were then stained with crystal violet. Both compounds provoke a remarkable reduction of cell migration with respect to the DMSO sample (Figure 9). To evaluate the degradation of extracellular matrix, Matrix Metalloproteinases 2 and 9 (MMP-2 and MMP-9) expression levels were investigated with Western Blot Analysis. The MMP-2 expression is significantly reduced when cells are treated with Cmp**5** with respect to the DMSO sample after both time points. After 24 h of treatment, the MMP-2 expression appears significantly increased in cells treated with Cmp**3** in respect to DMSO and Cmp**5** samples (Figure 10A,B). In parallel, the MMP-9 protein level is markedly augmented in cells treated for 6 h with Cmp**3** compared to Cmp**5** and DMSO samples. After 24 h of treatment, the MMP-9 expression was markedly reduced in cells treated with both compounds in respect to the DMSO sample (Figure 10 A,C). In addition, Cmp**3** provokes a marked reduction of MMP-9 from 6 to 24 h treatment, whereas Cmp**5** downregulates both MMPs (Figure 10A–C).

## 3. Discussion

It is widely reported in scientific literature that the MAO-B activity appears to increase with aging as well as in patients affected by neurodegenerative conditions including Parkinson’s disease and Alzheimer’s disease, thus explaining the administration of MAO-B inhibitors to treat the abovementioned diseases [10]. Nevertheless, Gabilondo and colleagues demonstrated a significant and selective increase in the MAO-B activity in human gliomas compared with non-tumoral tissues [5,11].

Therefore, we evaluated for the first time the biological effects of two novel MAO-B inhibitors, which were synthesized in our medicinal chemistry laboratory, on the C6 glioma cell line and on CTX TNA2 non-tumoral astrocytes, in terms of cell proliferation, apoptosis occurrence, inflammatory events and cell migration in order to improve the design and the assessment of anti-glioma therapies.

The two novel MAO-B inhibitors, Cmp**3** and Cmp**5**, appeared as promising molecules targeting MAO-B and reporting in glioma cells a percentage of cell viability notably lower than that recorded for TMZ, which is the most used drug in GBM. Surprisingly, we also found that Cmp**3** and Cmp**5** were capable of blocking tumor cell proliferation while sparing normal astrocytes. We further investigated this aspect by flow cytometric Annexin-V assay discovering that the block of tumor cell proliferation was unrelated to apoptosis or to necrosis.

As commonly reported in scientific literature in different experimental models of glioma, several cytotoxic drugs provoked an anti-proliferative effect inducing the G1 or G2/M cell cycle phase accumulation leading to a final cell cycle arrest [12,13]. Based on this knowledge, the cell cycle phases were measured by flow cytometry to further elucidate the biological mechanism underlying MAO-B inhibitory activity in tumor cells. We found that our novel MAO-B inhibitors, especially Cmp**5**, were responsible for an accumulation of C6 cells in the G1 phase and in parallel to a notable reduction of cells in the S phase. Our data are in accordance to previous studies demonstrating that MAO inhibitors, both MAO-A and MAO-B inhibitors, as well as pargyline and tranylcypromine, are able to inhibit the proliferation of prostate and breast cancer cells [14,15].

Furthermore, we measured the ROS production leading us to assume that the ROS increase after treatment with both MAO-B inhibitors could be largely responsible for the reduction of the S phase of C6 glioma cells, as already hypothesized [16]. It is possible to assume that our data on cell cycle report a clear temporal distinction between the ROS induction by Cmp**3** and Cmp**5**. Indeed, Cmp**5** is an early ROS inducer, whereas Cmp**3** is a stronger, but late inducer. This could represent a valuable and promising result considering that the increase of intracellular ROS can be therapeutically exploited. In fact, several chemotherapeutic agents may exert a selective toxic activity against cancer cells because they raise oxidative stress forcing the already stressed cells behind their limit [17]. Given that a notable reduction of the MMP predicts a marked oxidative stress, our data are further supported by the MMP, which appears markedly depolarized when the two MAO inhibitors are administrated. This trend could be related to the chemical-physical differences between the two compounds. Cmp**3** has a CLogP value of 4.30, whereas Cmp**5** has a CLogP value of 4.09, thus reinforcing our data on the early mitochondrial membrane depolarization possibly due to the Cmp**3** higher lipophilicity (associated to a better membrane permeability). The ability of our tested compounds to increase the base level of oxidative stress is also confirmed by the augmented PGE-2 secretion level measured after 24 h and 72 h of treatment, thus underlining the critical role played by oxidative stress in the mechanism of action of chemotherapeutic agents, as also hypothesized by Sun and colleagues [18].

Since it is well known that NOS activation is regulated by ROS and Reactive Nitrogen Species and that NOS-2 is implicated in human malignant tumors [19,20], we investigated the NOS expression. At an early stage, both our compounds did not modify the expression level of constitutive subtype of NOS-1, whereas they evidenced a remarkable ability to upregulate the expression level of inducible isoform NOS-2, thus contributing to the dramatic induction of a fatal drug-induced oxidative stress. However, the tested compounds did not reveal an up-regulation of NOS-2 at long time exposure, this could be due to a possible cell mechanism of resistance triggered after a longer drug administration.

Malignant gliomas are also well known for high vascularization suggesting that the recruitment of VEGF, the most important contributor of angiogenesis, represents a critical step for the progression of this malignancy [21]. Given that the VEGF expression is Nitric Oxide dependent [22], we evaluated the protein level, finding as expected, that the signaling pathway triggered by MAO-B inhibitors, involving ROS and NOS-2, is also supported by VEGF recruitment. It appears significantly augmented only at an early stage with Cmp**3**, thus supporting the hypothesis of the oxidative stress-promoted mechanism of action.

Moreover, the acquisition of a migratory phenotype is the prerequisite for its metastatic spreading and invasive potential [23]. Based on this evidence, we evaluated the effects of Cmp**3** and Cmp**5** on C6 cell migration. Surprisingly, both novel MAO-B inhibitors markedly reduced glioma cell migration, thus significantly preventing the invasiveness of brain tumors and their ability to infiltrate the neighbouring tissues as already demonstrated for several anti-glioma agents [24,25]. To clarify the molecular mechanism underlying this result, we evaluated the expression levels of MMP-2 and MMP-9, two proteases of the Matrix Metalloproteinase family involved in the remodeling and turnover of local extracellular matrix components. The MMP-2 and MMP-9 received particular attention given that their increased expression appeared related to the malignant potential of several types of cancer, including human gliomas [26]. We evidenced that Cmp**5** notably reduces glioma cell migration via the down-regulation of MMP-2 and MMP-9. These results are in agreement with data reported in scientific literature such as those reported by Webb [27] who explained that an up-regulation of MMP-2 and MMP-9 may result in cellular invasion, migration and in cancer metastasis. On the other hand, Cmp**3**, downregulating MMP-9 only at long time exposure, led us to assume that an alternative pathway could be involved in the reduction of cell migration.

In conclusion, this study demonstrates that our novel MAO-B inhibitors are able to contrast glioma proliferation by arresting the cell cycle and drastically increasing oxidative stress conditions, as well as the invasiveness by markedly reducing the migration of malignant cells. These findings reinforce the critical role played by MAO-B in mediating oncogenesis in high-grade glioma. Therefore, targeting the MAO-B protein could be a new approach to achieve improved therapeutic efficacy for glioblastoma.

## 4. Materials and Methods

### 4.1. Drugs and Reference Inhibitors

The two compounds were synthesized, purified and characterized as recently reported [6], whereas the two approved hMAO-B inhibitors (deprenyl and safinamide) were provided by Sigma-Aldrich (Milan, Italy). CLogP values for the newly-synthesized compounds were generated by ChemBioDraw Ultra 12.0.

### 4.2. Cell Lines

C6 rat glioma cell line (ECACC 92090409, Sigma Aldrich, Milan, Italy) and CTX/TNA2 rat astrocytes (ATCC CRL-2006TM) were cultured in Ham’s F12 and in DMEM high Glucose medium, respectively, supplemented with 10% of foetal bovine serum (FBS), 1% of penicillin/streptomycin and 1% of l-glutamine (all purchased by EuroClone, Milan, Italy). Cell cultures were maintained in an incubator in a humidified atmosphere with 5% CO_2_ at 37 °C.

### 4.3. MTT Assay

The C6 and CTX/TNA2 cells were seeded at cell density of 8000/well into a 96-well tissue culture plate. The metabolic activity of C6 and CTX/TNA2 cell lines was evaluated after 24 h and 72 h of treatment with Cmp**3** and Cmp**5** at 3.125, 6.25, 12.5, 25, 50, 100 μM and with TMZ at doses ranging from 15.625 up to 500 μM, on a 96-well polystyrene plate through MTT (3-[4,5-dimethyl-thiazol-2-yl]-2,5-diphenyltetrazolium bromide) assay (Sigma Aldrich, Milan, Italy). The assay is based on the capability of viable cells to reduce MTT into a colored formazan product. Compounds were dissolved in DMSO with a final concentration of 0.2%. At the established time points, the medium was replaced by a new one containing 0.5 mg/mL MTT and probed with cells for 4 h at 37 °C. The plate was incubated in DMSO solution for 30 min at 37 °C to solubilize salts and then read at 540 nm by means of a microplate reader (Multiskan GO, Thermo Scientific, MA, USA). Values obtained in the absence of cells were considered as background. Viability was normalized to control cells treated with the vehicle DMSO.

### 4.4. Detection of Apoptosis by Flow Cytometry

C6 rat glioma cells (2.375 × 10^5^/well) were cultured in 6-well plates for 24 h at 37 °C in a humidified atmosphere. The growth medium was then removed and the cells were subsequently exposed to treatments. Apoptosis detection was performed after 24 and 48 h collecting supernatants in tubes, cells were washed once with PBS at room temperature. Next, cells were trypsinized and collected by centrifugation together with supernatants. Apoptotic and necrotic cells were detected after staining cells with an Annexin-V and Propidium Iodide (PI) kit (eBioscience, Thermo Fisher Scientific, MA, USA), following the manufacturer’s instructions. Briefly, samples were incubated in 197 μL of binding buffer and 3 μL of Annexin-V FITC for 15 min at room temperature in the dark. Volumes were afterwards doubled, cells were washed once by centrifugation and re-suspended into 300 μL of binding buffer with PI. The fluorescence was determined by a CytoFlex flow cytometer (Beckman Coulter, CA, USA). FITC fluorescence (FL-1) was analyzed by a 530/30 band pass filter, and PI fluorescence (FL-3) by a 650 nm long pass filter. Data acquisition (2 × 10^4^ events/sample) and data analysis were performed with the CytExpert Software (Beckman Coulter, CA, USA). The percentages of viable cells (Annexin V−; PI−) were detected in the lower left quadrant (unstained) of density plots, as well as the cells in apoptosis (Annexin V+/PI−, lower right quadrant), late apoptosis (Annexin V+/PI+, upper right quadrant) and necrosis (Annexin V−/PI+, upper left quadrant).

### 4.5. Cell Cycle Analysis

The C6 rat glioma cells were seeded and exposed as previously described in this section. After 24 h exposure, the medium was removed and cells were washed once with PBS, trypsinized and collected by centrifugation. Next, the cells were counted by means of Trypan blue dye exclusion test and 3 × 10^5^ cells/sample were fixed in cold ethanol 70% v/v and kept at 4 °C overnight. After that, the cells were gently washed with cold PBS and centrifuged at 2700 g for 10 min at 4 °C. After having discarded supernatants, each sample was incubated in 300 μL of the staining solution containing PBS without calcium and magnesium, RNase 100 μg/mL (stock solution 10 mg/mL in 10 mM sodium acetate buffer, pH 7.4) and PI 10 μg/mL (stock solution 1 mg/mL in water) (all purchased by Sigma Aldrich, MI, USA) and kept at 4 °C overnight in the dark. PI fluorescence was detected by a flow cytometer equipped with a 488 nm laser (CytoFlex flow cytometer, Beckman Coulter, CA, USA) in the FL-3 channel (620 nm of wavelength emission). 2 × 10^4^ events/sample were collected and analyzed with CytExpert Software (Beckman Coulter, CA, USA). The percentage of cells in G1, S, or G2 phase of the cell cycle were calculated after mathematical modeling of histograms using the FCS Express Flow Cytometry Data Analysis (De Novo Software, CA, USA).

### 4.6. Flow Cytometry Analysis of ROS and MMP

The C6 rat glioma cells were cultured and stimulated as described for the apoptosis assay. The intracellular generation of ROS was determined using the chloromethyl derivative of 2′,7′-Dichlorofluorescein Diacetate (DCF-DA) (CM-H2DCF-DA, Molecular Probes, Invitrogen, CA, USA), an oxidation-sensitive probe, while the fluctuations of the mitochondrial membrane potential was monitored by means of the TMRE (Tetramethylrhodamine, Ethyl Ester) Assay Kit (Abcam, Cambridge, UK). After 6 and 24 h, exposure medium was removed and cells were incubated with PBS/FBS 10% and CM-H2DCFDA 2.5 μM at 37 °C in a humidified atmosphere in the dark. After 30 min, 100 nM TMRE was added and cells were incubated for an additional 30 min. Next, cells were washed once with PBS, trypsinized and collected by centrifugation. After having resuspended cells in 30 μL of PBS, DCF and TMRE fluorescence emissions were measured by flow cytometry (CytoFlex flow cytometer, Beckman Coulter, CA, USA) at an excitation wavelength of 488 nm and an emission wavelength of 527 nm (DCF-FITC) and 575 nm (TMRE-PE). Mean Fluorescence Intensity (MFI) was obtained by histogram statistics using the CytExpert Software (Beckman Coulter, CA, USA). Individual values of MFI were normalized on negative controls (unstained samples) and are provided as mean values of MFI ratio ± SD.

### 4.7. Western Blot Analyses

The C6 cell lysates (20 µg) were electrophoresed and transferred to the nitrocellulose membrane. Nitrocellulose membranes, blocked in 5% of non-fat milk in PBS 0.1% Tween-20, were probed with mouse monoclonal anti-β actin antibody (antibody dilution 1:5000) (A5316 Sigma, MO, USA), rabbit polyclonal anti-VEGF anti-NOS-2 antibodies (antibodies dilution 1:200) (sc-152, sc-651, respectively, both purchased by Santa Cruz biotechnology, CA, USA), mouse monoclonal anti-HIF-1α, anti-MMP-9, anti-MMP-2, anti-NOS-1 antibodies (antibodies dilution 1:200) (sc-5354, sc-393859, sc-13595, sc-5302, respectively, all purchased by Santa Cruz biotechnology, CA, USA), then incubated in the presence of specific enzyme conjugated IgG horseradish peroxidase. Immunoreactive bands were detected by ECL system (Amersham Int., Buckunghamshire, UK) and analyzed by densitometry. Densitometric values, expressed as Integrated Optical Intensity (IOI), were estimated in a CHEMIDOC XRS system by the QuantiOne 1-D analysis software (BIORAD, Richmond, CA, USA). Values obtained were normalized based on densitometric values of loading control β-actin.

### 4.8. ELISA Test for PGE-2

The PGE-2 secretion in the culture medium was detected by following the instructions provided by the manufacturer’s protocol. EIA kit (Enzo Life Sciences, Farmingdale, NY, USA) was used to measure PGE-2 concentrations. The absorption values were obtained by the spectrophotometric reading of plates at 450 and 405 nm, respectively, by means of a microplate reader (Multiskan GO, Thermo Scientific, MA, USA). Secretion levels of PGE-2 were analyzed in different wells after treatment with Cmp**5** at 50 μM and Cmp**3** at 100 μM, normalized for relative optical density (pg/mL/OD) as previously determined by the MTT assay.

### 4.9. Transwell Migration Assay

The C6 cell migration was evaluated by means of a 24-well Transwell Boyden chamber containing 8 µm pore size membranes (Corning, Lowell, MA, USA). For this purpose, suspended C6 rat glioma cells were separately treated with 50 µM Cmp**5** and 100 µM Cmp**3** in serum-free Ham’s F12 at cell density of 50,000/150 μL, and then added to the upper chamber of a 8 μm pore size insert. Ham’s F12 supplemented with 10% FBS was added to the lower chamber as a chemoattractant and allowed to migrate towards a 10% FBS containing medium present in the lower chamber. Cells were incubated for 24 h at 37 °C, the non-migrating cells on the upper chamber were then removed with the aid of a cotton swab, whereas the cells migrated to the lower surface of the membrane were stained with crystal violet for 10 min at room temperature. Images were captured at 20x magnification with a light microscope (Leica DM 4000, Leica Cambridge Ltd., Cambridge, UK) equipped with a Leica DFC 320 camera (Leica Cambridge Ltd.). Images were analyzed by means of Leica Application Suite–X (LAS-X) analysis software. The migration rate was calculated as the area covered by stained cells, given that they represent the migrated cells.

### 4.10. Statistics

Statistical analysis was executed with GraphPad 7 software using t-test and Ordinary One-Way ANOVA followed by post-hoc Tukey’s multiple comparisons tests. Values of *p* <0.05 were considered statistically significant.

## Supplementary Materials

The following are available online. Figure S1: MTT cell viability assay on C6 and CTX TNA2. (A, C) Histograms represent the viability dose-response of C6 cells exposed to different concentrations of deprenyl and safinamide (0.625 up to 10 μM) for 24 h and 72 h. (B, D) Histograms represent the viability dose-response of CTX cells exposed to different concentrations (0.625 up to 10 μM) of deprenyl and safinamide for 24 h and 72 h. Proliferation was assessed using MTT assay and normalized to control cells treated with DMSO (0.2% as final concentration).
