# Supplementary material for: The Up-Regulation of Oxidative Stress as a Potential Mechanism of Novel MAO-B Inhibitors for Glioblastoma Treatment
Source: Molecules. 2019 May 25;24(10):2005. doi: 10.3390/molecules24102005 (PMC6572653; doi:10.3390/molecules24102005)
Supplement: Supplementary file 1 [file molecules-24-02005-s001.pdf]

Supplementary material

The Up-Regulation of Oxidative Stress as a Potential Mechanism of Novel MAO-B Inhibitors for Glioblastoma Treatment

Guya Diletta Marconi [1], Marialucia Gallorini [1], Simone Carradori [1,*], Paolo Guglielmi [2], Amelia Cataldi [1] and Susi Zara [1]

[1] Department of Pharmacy, University "G. d'Annunzio" of Chieti-Pescara, Via dei Vestini 31, 66100 Chieti, Italy; guya.marconi@virgilio.it (G.D.M.); marialucia.gallorini@unich.it (M.G.); simone.carradori@uniroma1.it (S.C.); amelia.cataldi@unich.it (A.C.); susi.zara@unich.it (S.Z.)

[2] Department of Drug Chemistry and Technologies, Sapienza University of Rome, P.le A. Moro 5, 00185 Rome, Italy; paolo.guglielmi@uniroma1.it

* Corresponding author: simone.carradori@uniroma1.it, Tel.: +39-0871-355-4583.

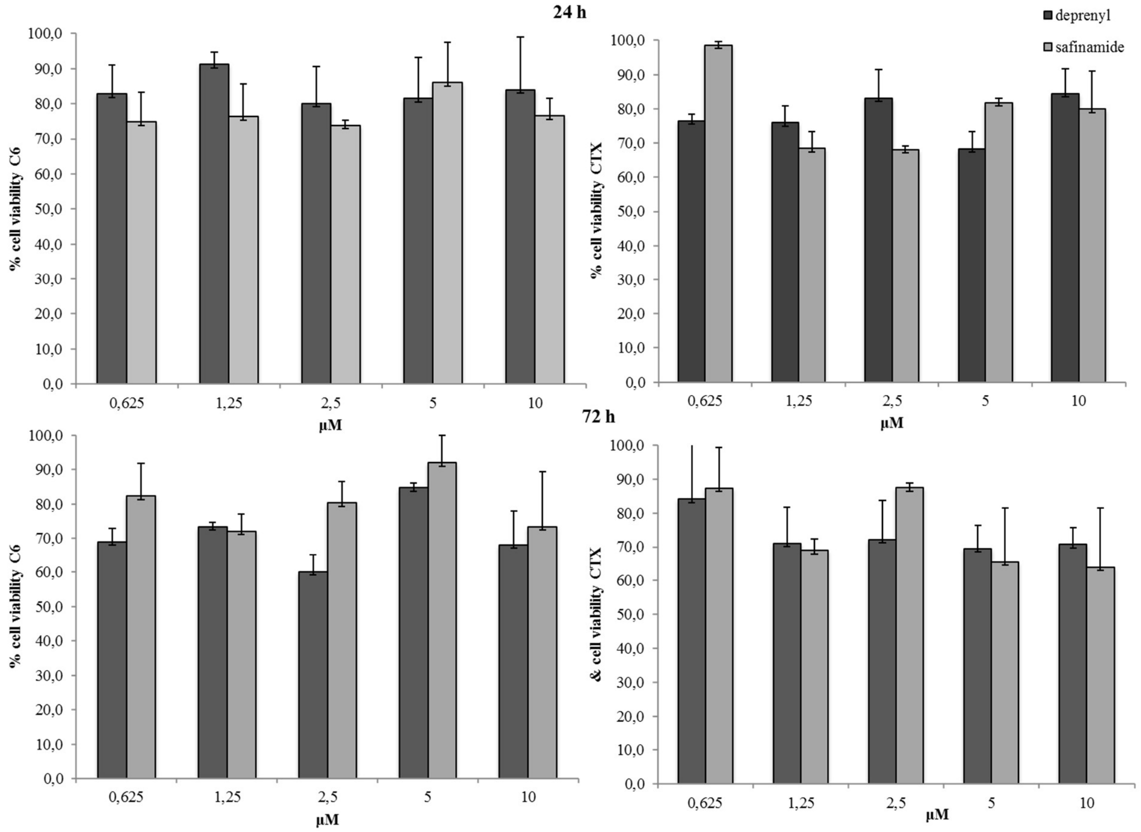

Figure 1. MTT cell viability assay on C6 and CTX TNA2. (A, C) Histograms represent the viability dose-response of C6 cells exposed to different concentrations of deprenyl and safinamide (0.625 up to 10 µM) for 24 h and 72 h. (B, D) Histograms represent the viability dose-response of CTX cells exposed to different concentrations (0.625 up to 10 µM) of deprenyl and safinamide for 24 h and 72 h. Proliferation was assessed using MTT assay and normalized to control cells treated with DMSO (0.2% as final concentration).
